# Liquid/liquid interface assisted *in situ* polymerisation of aniline on Ti_3_C_2_T_*x*_ MXene for electrochemical detection of dopamine†

**DOI:** 10.1039/d5na00374a

**Published:** 2025-07-08

**Authors:** Anjali Sugunan, Aiswarya Anil Syamala, Athul Beena Radhakrishnan, Mini Mol Menamparambath

**Affiliations:** a Department of Chemistry, National Institute of Technology Calicut Calicut 673601 Kerala India minimol@nitc.ac.in neeharabindu@gmail.com

## Abstract

The liquid/liquid (L/L) interface-assisted polymerisation technique, unlike bulk or single-phase polymerisation, has the potential to offer effective control of the self-assembly and diffusion of reactive intermediates and versatile tuning of the morphology at the interface to allow tailored properties within the functional nanostructures. This study adopts an *in situ* L/L interface-assisted polymerisation approach to generate Ti_3_C_2_T_*x*_ MXene/PANI with enhanced electrochemical characteristics. The aniline released at the L/L interface in a controlled manner interacts with the inherent negative charge of MXene, initiating an *in situ* polymerisation of PANI over the surface and interlayers of MXene to yield hydrophilic MXene/PANI nanostructures. Furthermore, their electrochemical properties are notably enhanced compared to those of hybrid structures formed *via in situ* single-phase polymerisation. The comprehensive research demonstrated that MXene/PANI formed at the L/L interface resulted in better exfoliation of the MXene due to the integration of fibrillar natured PANI, whereas the MXene was encased by aggregated PANI structures during single-phase polymerisation. The advancement of reactant consumption and product formation in the corresponding organic/aqueous phases was monitored using UV-visible spectroscopy, indicating controlled polymerisation at the L/L interface. The controlled release of reactants *via* interface circumvents side products or undesirable side-chain branching reactions, leading to the *in situ* generation of long-chain polymers. The successful intercalation of PANI into the interlayers of MXene was evident from physicochemical investigations such as Raman, XRD, SEM, and HRTEM. The MXene/PANI composites generated by *in situ* L/L interface-assisted polymerisation offered excellent electrochemical performance compared to the *in situ* single-phase polymerisation method. Ultimately, the synthesised nanohybrid Ti_3_C_2_T_*x*_ MXene/PANI-modified GCE demonstrates enhanced non-enzymatic DA sensing capabilities with a detection limit of 34 nM.

## Abstract 2

Investigation of the *in situ* L/L interface-assisted polymerisation approach for generating Ti_3_C_2_T_*x*_ MXene/PANI with enhanced electrochemical characteristics.

## Graphical abstract

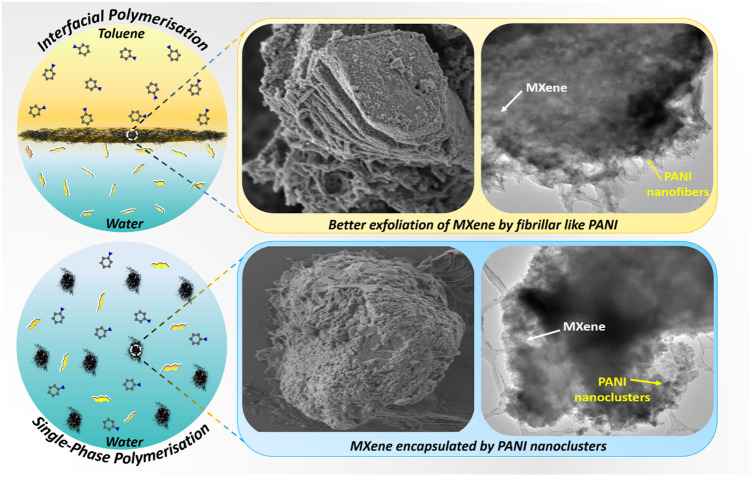

## 1. Introduction

MXenes, a family of transition metal carbides and nitrides, have garnered attention due to their distinctive features, including great chemical stability, exceptional electrical conductivity, extensive surface area, biocompatibility, and facile dispersion in polar solvents.^1–4^ These attributes make MXenes an intriguing alternative for applications in electrochemical energy storage,^5–8^ sensing,^9–11^ nanogenerators,^12,13^ electromagnetic interference (EMI) shielding,^14,15^ and photothermal conversion.^16–18^ MXenes, unlike many conventional 2D materials, retain outstanding electrical conductivity even at the monolayer level and possess tunable physicochemical properties enabling rapid and efficient electron transfer at the interface between the sensor and analyte, making them highly promising materials for electrochemical sensing applications.^19,20^ Nevertheless, pristine MXenes exhibit comparatively limited electrocatalytic performance, charge transfer efficiency, and a propensity to restack owing to van der Waals forces and instability under aqueous conditions.^21–23^ The restacking of MXene restricts the accessibility of active sites and the flow of electrolyte ions, negatively impacting its electrochemical performances. Diverse studies have been undertaken to address these fundamental constraints of MXene for achieving improved electrocatalytic activity.^24,25^

The development of MXene-based hybrids through the integration of pristine MXene with metal nanoparticles,^26–28^ metal oxides,^29,30^ conducting polymers,^31,32^ or carbon materials^23,33^ presents a promising solution to the challenge of layer restacking, while retaining the excellent electrical conductivity of MXene. This strategy also helps shield MXenes from oxidative degradation by reducing their direct exposure to oxygen.^2^ These engineered nanostructures allow for fine-tuning of electrochemical behaviour, making them highly effective for the targeted detection of analytes and paving the way for more sensitive, selective, and durable electrochemical sensors.^34,35^ Intercalating interlayer spacers between MXene layers is a successful strategy to solve these issues.^36^ “Organic modifiers” are an ideal candidate for serving as efficient interlayer spacers for MXenes, allowing the development of hybrid nanostructures. On top of that, the distinctive surface and interface engineering of MXene nano-architectures results in subsequent generations of active surfaces abundant in functional groups such as O, OH, and F, which serve as nucleation sites and substrates for polymers.^36,37^ The MXene-polymer interactions, influenced by electrostatic forces, van der Waals interactions, and hydrogen bonding, significantly improve electrocatalytic performance and stability, making them beneficial for electrochemical applications.^38,39^

Among the various approaches employed to achieve MXene/polymer nanocomposites, *in situ* polymerisation on the surface of MXene emerges as a particularly promising technique for creating composites that effectively integrate MXene with polymers.^40^ The *in situ* polymerisation process begins with the organisation of monomers on the MXene surface and the intercalation into the interlayers of MXene through hydrogen bonds and/or electrostatic interactions, which is then followed by polymerisation to establish polymer coverage.^31,41^ Nonetheless, these established processes reveal considerable shortcomings. The primary constraint lies in the difficulty of precisely managing the morphology and extent of deposition during *in situ* polymerisation, resulting in a non-uniform polymer coating, which negatively impacts the electrochemical performance of the electrodes.^36,42,43^ Moreover, managing the impacts of polymer loading and the thickness of the deposited polymer during *in situ* polymerisation poses considerable challenges. The efficacy of Ti_3_C_2_T_*x*_/polymer composites can be attained solely when the polymer exhibits uniform growth on the layered architecture of Ti_3_C_2_T_*x*_.^44^ Furthermore, controlling the amount of polymer applied to the surface of MXene facilitates the formation of electrodes that exhibit improved ion transport properties and electrochemical performance. The abundant hydrophilic functional groups present in polyaniline (PANI) skeletons guarantee advantageous wettability, thus enhancing the ion-accessible specific surface area and promoting charge transfer.^42,45,46^ As a result, PANI can be meticulously engineered and assembled as a promising “organic modifier” to enhance the surface of Ti_3_C_2_T_*x*_ MXene, thereby advancing the electrochemical properties of the composites and augmenting their practical applications.^47–49^ The combined influence of the metallic conductivity inherent in Ti_3_C_2_T_*x*_ and the remarkable redox properties of PANI can enhance the efficacy of an electrochemical sensor and thus represent a successful strategy for the design of high-performance electrodes.^23,27^ Unlike single-phase polymerisation, such as dilute and electrochemical polymerisation methods, which produce MXene/polymer nanocomposites that feature aggregated and coarser MXene structures, the interfacial polymerisation reaction is initiated at a deliberate and measured pace.^50^ The latter process is facilitated by the selective and controlled diffusion of monomers towards the interface, driven by unbalanced forces,^42,51^ ultimately resulting in MXene/polymer nanostructures that exhibit relatively uniform and conformal distribution of polymer across the MXene surface and within its interlayers.

In this study, we report an enormously simple, single-step approach for the controlled synthesis of MXene/PANI nanocomposites by a L/L interface-assisted *in situ* polymerisation method for the development of MXene/PANI electrodes, demonstrating improved electrochemical performance towards sensing of dopamine (DA). DA is a vital monoamine neurotransmitter and neuromodulator involved in a broad range of physiological processes, particularly within the central nervous system, where it influences cognition, emotion, motor control, and cardiovascular and renal functions.^52^ Imbalances in DA levels are linked to major health conditions, with low levels associated with Parkinson's disease, Alzheimer's disease, and depression, and elevated levels linked to schizophrenia and psychosis.^53,54^ Given the clinical relevance of DA, its accurate and dependable quantification is imperative for the early diagnosis and effective management of related disorders.^55^ While traditional analytical techniques provide high sensitivity, their application is often limited by high operational costs, complex procedures, and the necessity for trained personnel.^56^ In contrast, the intrinsic electrochemical activity of DA enables its direct detection through non-enzymatic electrochemical sensing, a method that offers notable advantages, including operational simplicity, rapid analysis, cost-effectiveness, and suitability for portable diagnostic platforms^35,57^

Aniline monomers dissolved in toluene have been employed as the organic phase, with a density lower than that of the aqueous phase, where the MXene and oxidant were distributed. During *in situ* generation, the MXene/PANI nanocomposite initially originated at the interface between two immiscible phases and subsequently migrated to the lower aqueous phase. Detailed investigations evidenced that the MXene/PANI generated employing the L/L interface-assisted polymerisation method resulted in better exfoliation of the MXene, followed by the integration of fibrillar natured PANI, whereas the MXene was encapsulated by aggregated PANI structures during single-phase polymerisation. The electrochemical analysis of both materials demonstrates that the MXene/PANI synthesised through the interface method functions as a more efficient electrode material in comparison to the single-phase nanocomposites. Furthermore, the controlled exposure of active MXene surfaces in MXene/PANI polymerised at the L/L interface led to better electrocatalytic activity towards the detection of DA. The foremost benefit of our methodology lies in its environmental friendly method for executing controlled polymerisation and *in situ* generation of the MXene/polymer nanocomposite showing enhanced electrocatalytic properties.

## 2. Experimental section

### 2.1 Chemicals and reagents

Titanium aluminium carbide (Ti_3_AlC_2_) MAX phase originally from Y Carbon Ltd.-Ukraine, hydrofluoric acid (HF) (Thermo Fisher Scientific India, 48%), hydrochloric acid (Thermo Fisher Scientific India, 36%), aniline (Alfa Aesar, 126213, 97%), toluene (Emplura, Merck Specialities Private Limited), ammonium per sulphate (APS) (Sigma Aldrich, 98%), potassium ferricyanide (K_3_[Fe(CN)_6_]) (Fisher Scientific), potassium chloride (KCl) (Fischer Scientific, 7447-40-7), sodium chloride (NaCl) (Nice, 99.9%), phosphate-buffered saline (pH 7.2, Himedia, 9004-65-3), dopamine hydrochloride (Sigma-Aldrich, 62-31-7, 98%), uric acid (Alfa Aesar, 69-93-2, 99%), l-ascorbic acid (Thermo Scientific, 50-81-7, 99%), (*S*)-(+)-glutamic acid (Sigma-Aldrich, 56-86-0), histamine dihydrochloride (Sigma-Aldrich, H7250-5G, 99%), glucose (Thermo scientific, 50-99-7, 99%), and phosphate-buffered saline (pH 7.2, Himedia, 9004-65-3) were of analytical grade and were used without further purification. Deionized (DI) water (ELGA Purelab Quest UV, 18.2 MΩ) was used throughout the experiment.

### 2.2 Synthesis of Ti_3_C_2_T_*x*_ MXene

Ti_3_C_2_T_*x*_ was synthesised by the conventional aluminium (Al) etching process of Ti_3_AlC_2_ MAX phase using 48% HF.^58^ Initially, 1 g of Ti_3_AlC_2_ is added slowly over 5 min into a 60 mL polypropylene bottle containing a solution mixture of 2 mL of 48% HF, 6 mL of DI water, and 12 mL of 12 M HCl. The Al etching process was continued for 24 h at 35 °C under magnetic stirring at 500 rpm. The resulting solution was then subjected to centrifugation at 6000 rpm for 30 min, followed by multiple washes with DI water until the supernatant achieved a neutral pH. Finally, the resulting sediment was collected and freeze-dried for 12 hours at −50 °C, resulting in Ti_3_C_2_T_*x*_ MXene. Schematics for the preparation of Ti_3_C_2_T_*x*_ MXene from the Ti_3_AlC_2_ MAX phase and the corresponding SEM morphologies are shown in Fig. S1.† The as-prepared Ti_3_C_2_T_*x*_ MXene is dispersed in water and sonicated to obtain a stable colloidal dispersion of MXene (0.1 g mL^−1^) for the *in situ* synthesis of the MXene/polymer composites.

### 2.3 *In situ* synthesis of MXene/polyaniline (MX/PANI) nanocomposites

#### 2.3.1 Using the L/L interface-assisted polymerisation technique

A bisolvent interface-assisted *in situ* oxidative polymerisation of aniline was employed for the synthesis of the MXene/PANI composite. 5 mmol of APS is dissolved in 100 mL of 2 M HCl, and to this acidic solution, a required amount of 0.1 g mL^−1^ of MXene dispersion is added. A 100 mL of toluene containing 5 mmol of aniline monomer is added carefully and spread over the MXene dispersion. After the complete addition, the two immiscible phases with respective precursor molecules form an interface, and the reaction medium is left under magnetic stirring in an ice bath (5 to 10 °C) at 100 rpm without interrupting the bisolvent interface. During the reaction, the interface can be regarded as the site of initiation, where the polymerisation starts instantly, and the dark green product gradually migrates into the lower aqueous layer (Fig. S2a†). After 12 hours of polymerisation reaction, the resultant product in the lower aqueous phase is collected by vacuum filtration and dried for 24 hours at 35 °C in a vacuum oven. The MXene/PANI nanocomposite synthesized by the interfacial approach is referred to as MX/PANI-IN throughout the work.

#### 2.3.2 Using the single-phase polymerisation technique

In contrast to interface-assisted polymerisation, all the constituents required for the polymerisation are retained in an aqueous medium alone while maintaining the same compositions and reaction conditions (Fig. S2b†). The MXene/PANI nanocomposite synthesized by the single-phase approach is referred to as MX/PANI-SI throughout the work. Further details on the synthesis parameters, composition, and sample codes of the MXene/PANI composites synthesised using the interface and single-phase polymerisation strategies are tabulated in the ESI (Table S1†). Pure PANI was also synthesized using the same two methodologies for comparative investigations.

### 2.4 Instrumentation

The morphology and composition analysis of the synthesized MXene/PANI nanocomposites was performed using a high-resolution scanning electron microscope (HRSEM, Zeiss) equipped with an energy dispersive spectrometer (Zeiss SmartEDX). Powder X-ray diffraction (XRD) studies were performed using a PANalytical X'Pert3 powder X-ray diffractometer with Cu Kα radiation. Fourier transform infrared (FTIR) spectroscopy studies were performed in KBr pressed pellets using a PerkinElmer Frontier MIR spectrometer. The crystal structure and atomic resolution imaging of the samples were investigated with a high-resolution transmission electron microscope (HR-TEM, TALOS F200S G2). The UV-visible analysis was performed using a Shimadzu UV-2600 spectrophotometer. The chemical makeup of the composites was analyzed using Raman spectroscopy (Invia Reflex Raman microscope with a spectrometer and a laser source with an excitation wavelength of 532 nm). The surface chemistry of the synthesized samples was analyzed using XPS (Thermo Fischer K-alpha) equipped with a monochromatic, micro-focused Al Kα X-ray source (*hν* = 100–4000 eV). Electrochemical measurements were performed using a CHI660E and Origalys OGF500 electrochemical workstations with a standard three-electrode system.

### 2.5 Electrode fabrication for electrochemical analysis

A glassy carbon electrode (GCE) with a diameter of 3 mm was polished with 0.05 μm alumina slurry, followed by sonication in ethanol and DI water for cleaning. Ag/AgCl (1 M KCl) acts as the reference electrode, and platinum (Pt) as the counter electrode. The GCE surface was activated by repeated CV scans in 0.5 M H_2_SO_4_ to provide steady voltammograms. For each measurement, 5 mg of the composites were ultrasonically suspended in 1 mL of distilled water to obtain a homogeneous suspension. 6 μL of this sample dispersion was transferred onto the pretreated glassy carbon electrode using a micropipette and dried under an infrared lamp to obtain the modified GCE. In the presence of 1 μM DA, CV analysis was performed using each modified GCE at applied voltages between −0.8 and + 0.8 V and a scan rate set to 100 mV s^−1^ in N_2_-saturated potassium phosphate buffer solution (PBS, 0.1 M, pH 7.2).

## 3. Results and discussion

### 3.1 Microstructural analysis

**Fig. 1:**
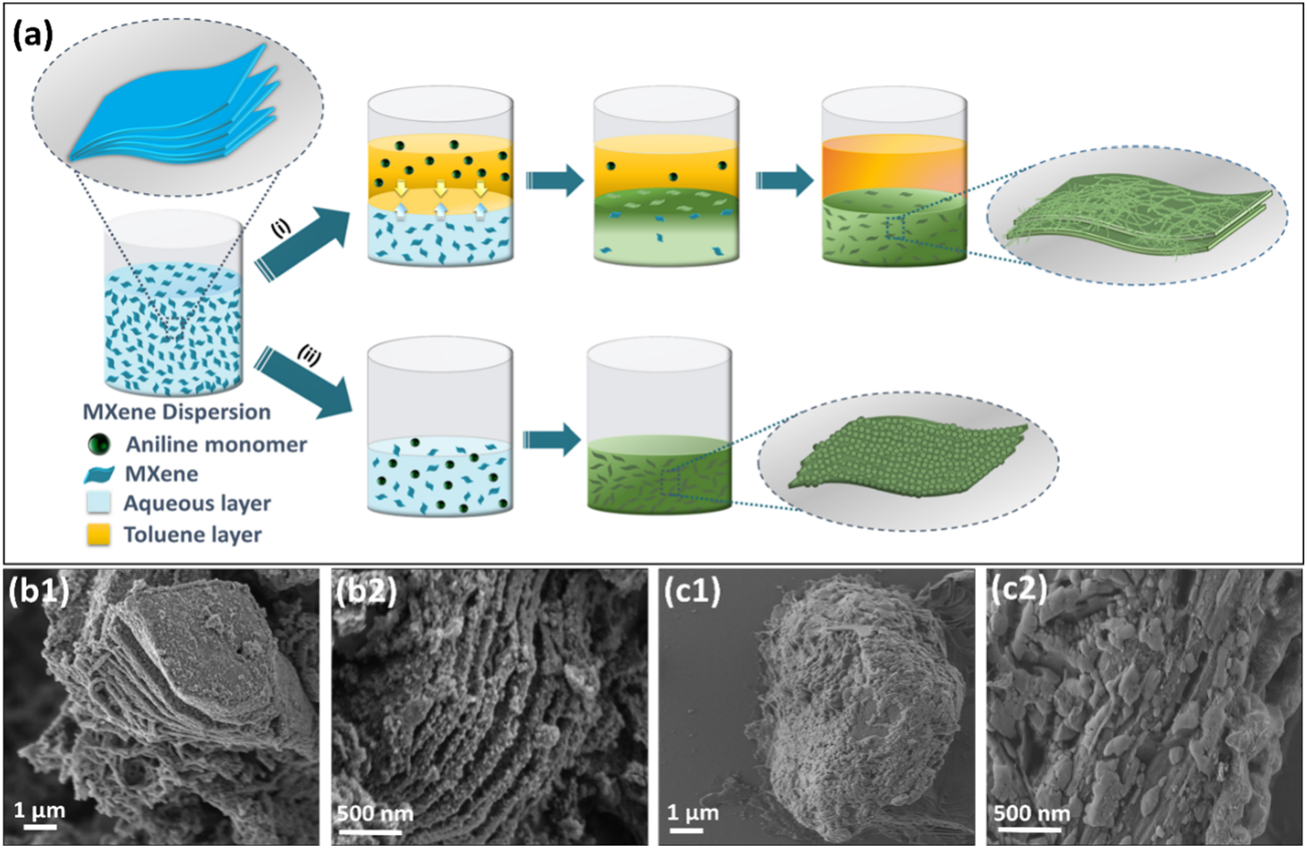
(a) Schematics of the two different synthesis strategies for the *in situ* generation of MXene/PANI nanocomposites: (i) L/L interface-assisted *in situ* polymerisation and (ii) single-phase *in situ* polymerisation; SEM images of MXene/PANI nanocomposites synthesized by (b1 and b2) the L/L interface method and (c1 and c2) the single-phase method.

The presence of terminal groups such as =O, –OH, and –F on the layered Ti_3_C_2_T_*x*_ could offer active electrophilic sites that can function as templates for polymerisation.^59^Fig. 1a demonstrates the schematic comparison of the *in situ* generation of MXene/PANI composites through the L/L interface-assisted and single-phase polymerisation strategies. Initially, Ti_3_C_2_T_*x*_ MXene, acquired by selective etching of Ti_3_AlC_2_ with HF, is dispersed in distilled water subjected to sonication for 10 minutes, resulting in a stable colloidal dispersion. For *in situ* polymerisation at the L/L interface, the aniline monomer is evenly distributed in toluene and is added as a top layer to the lower acidic solution containing HCl, Ti_3_C_2_T_*x*,_ and dissolved APS (Fig. 1a(i)). Consequently, an interface is conceptualized as an abstract membrane that initiates the selective flow of reactive molecules between the two immiscible phases.^60^ The interface facilitates polymerisation *via* the self-assembly and nucleation of monomers, as well as the selective transport of monomers and reactants from their respective phases towards it.^51,61^ The diffusion of aniline monomers transpires at a controlled rate towards the interface, where they interact with the protic acid located in the lower aqueous phase. This interaction results in the transformation of the monomers into aniline radical cations at the interface. Simultaneously, electrostatic interactions between the positively charged aniline radical cations and the negatively charged Ti_3_C_2_T_*x*_ surface groups facilitate the adsorption of aniline cationic radicals on the surface as well as intercalation into the interlayers of Ti_3_C_2_T_*x*_ MXene. This subsequently triggers the polymerisation of aniline cations; in the presence of APS, the mechanism of aniline polymerisation is provided in Fig S3.^48,61^† The insertion and anchoring of PANI chains occur *in situ* on the surface of MXene nanosheets through profound chemical interactions involving residual oxygen-containing functional groups, as well as van der Waals forces and hydrogen bonding interactions.^42^ This process culminates in the formation of distinctive tubular structured PANI-decorated MXene nanosheets in a regulated manner *via* a self-assembly mechanism.^62^ The possible better interaction of MXene/PANI towards the lower aqueous phase when compared with that toward toluene or the interface, leads to the accumulation of the products in the aqueous phase. The PANI nanostructures, created *via* interfacial polymerisation, are not subjected to further polymerisation, resulting in MXene/PANI nanostructures characterized by controlled PANI deposition and intercalation on their surface and interlayers, respectively. The interactions of reactive intermediates at the L/L interfaces differ completely from those of a single-phase reaction mixture (Fig. 1a(ii)) where all of the reactive components are in the same phase, resulting in aggregated non-uniform PANI deposition on the surface of MXene with no accurate control of polymerisation. The microstructures of the Ti_3_AlC_2_ MAX phase, Ti_3_C_2_T_*x*_ MXene, and MXene/PANI nanocomposites synthesized by the interface and single-phase polymerisation methods was investigated by SEM. The regularly aligned lamellar structure of multilayer Ti_3_C_2_T_*x*_ MXene (Fig. S1c†) reveals that the Al layer was successfully removed by HF etching of the Ti_3_AlC_2_ MAX precursor (Fig. S1b†).

As illustrated in Fig. 1(b1) and (b2), it was observed that tubular PANI with a textured surface was distributed uniformly across the interlayers and surface of MXene nanosheets, causing a better exfoliated lamellar structure and creating an open and interlocking architecture that could proficiently counteract the stacking and collapsing of MXene nanosheets. Furthermore, the rough surface of PANI offers a multitude of active sites in interaction with electrolyte ions, thereby facilitating electrolyte accessibility, accelerating ion diffusion, and improving electrochemical performance.^63^ The SEM images of MX/PANI-SI in Fig. 1(c1) and (c2) depict a dense accumulation of PANI completely covering the lamellar structure of MXene. Furthermore, the SEM images confirm that controlled polymerisation offered by the L/L interface is advantageous over single-phase polymerisation in exfoliating the MXene layers through an *in situ* polymerisation strategy. The distribution of elements in the composite was studied by SEM elemental mapping through energy-dispersive spectroscopy (EDS). The elemental maps (Fig. S4†) of MX/PANI-IN (a–e) and MX/PANI-SI (f–j) confirm PANI incorporation on MXene. MX/PANI-IN shows a controlled and well-integrated distribution of C, N, and Cl, while MX/PANI-SI exhibits denser and less controlled PANI integration.

**Fig. 2:**
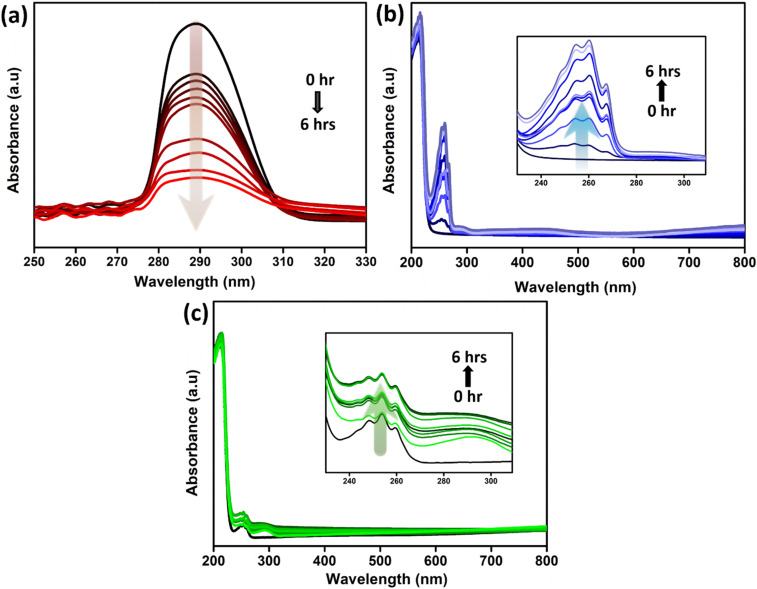
UV-visible spectra recorded at regular intervals of 0, 0.5, 1, 1.5, 2, 3, 3.5, 4, 5, and 6 hours during *in situ* generation of MX/PANI-IN composites at the interface. Characteristic absorptions from (a) the low-density upper toluene layer and (b) the lower aqueous layer, and the inset shows the magnified region of the spectra from 230 to 350 nm. (c) UV-visible spectra recorded for MX/PANI-SI formation (single-phase polymerisation) at regular intervals of 0, 0.5, 1, 1.5, 2, 3, 3.5, 4, 5, and 6 hours (the inset shows the magnified region of the plot from 230 to 350 nm).

The progression of the consumption of reactants and the appearance of products in the respective phases during polymerisation was monitored using UV-visible spectroscopy, with spectra collected at regular intervals of 0, 0.5, 1, 1.5, 2, 3, 3.5, 4, 5, and 6 hours. For each analysis, 100 μL of the reaction mixture was collected from the respective phases during the progress of the reaction and diluted to 3 mL in a quartz cuvette to obtain the UV-visible spectra. Fig. 2a and b show the UV-visible absorption spectra recorded from the upper toluene layer and the lower aqueous layer, respectively, during interfacial polymerisation. For recording the absorbance spectra, samples were collected from areas proximal to the interface. As can be seen in Fig. 2a, pure aniline in the toluene phase shows a broad absorption peak at 288 nm due to the π–π* transition of the benzenoid structure.^64,65^ Whereas, aniline radical cations and their oligomers exhibit UV absorbance at lower wavelengths in the range of 230 to 300 nm^64–66^ due to resonance stabilization. During the initiation, aniline comes in contact with APS under aqueous acidic conditions at the interface, which in turn converts aniline to a more polar aniline radical cation as illustrated in Fig. S3.† The initial sharp decline in absorption intensity of aniline indicates the diffusion of the aniline radical cation towards the lower aqueous phase, due to the initiation of the polymerisation reaction. During the course of the reaction, aniline monomers are consumed from the organic layer to generate oligomers and polymers successively, resulting in a decrease in the concentration of aniline monomers. Concomitantly, Fig. 2b illustrates an increase in the intensity of the UV-visible absorption spectra in the range of 230 to 300 nm of the lower aqueous phase. The emergence of new absorption bands within the spectral range of 430 to 450 nm corresponds to the emeraldine oxidation state of polyaniline, indicating the formation of polymeric chains as shown in Fig. S5(a).†

The progression of the consumption of reactants and the appearance of products during polymerisation was also monitored in single-phase polymerisation (Fig. 2c). Notably, the emergence of dimeric or oligomeric peaks occurred instantly, as evident from the absorbance spectra at 0 hours. However, as can be observed in Fig. S5(b),† polymeric peaks are absent in single-phase polymerisation, highlighting the specific challenge in regulating the reaction conditions to achieve the desired long-chain polymers that favour the intended linear head-to-tail aniline polymerisation. It should be noted that the controlled release of reactants *via* the interface circumvents side products or undesirable side-chain branching coupling reactions, leading to the *in situ* generation of long-chain polymers.

**Fig. 3:**
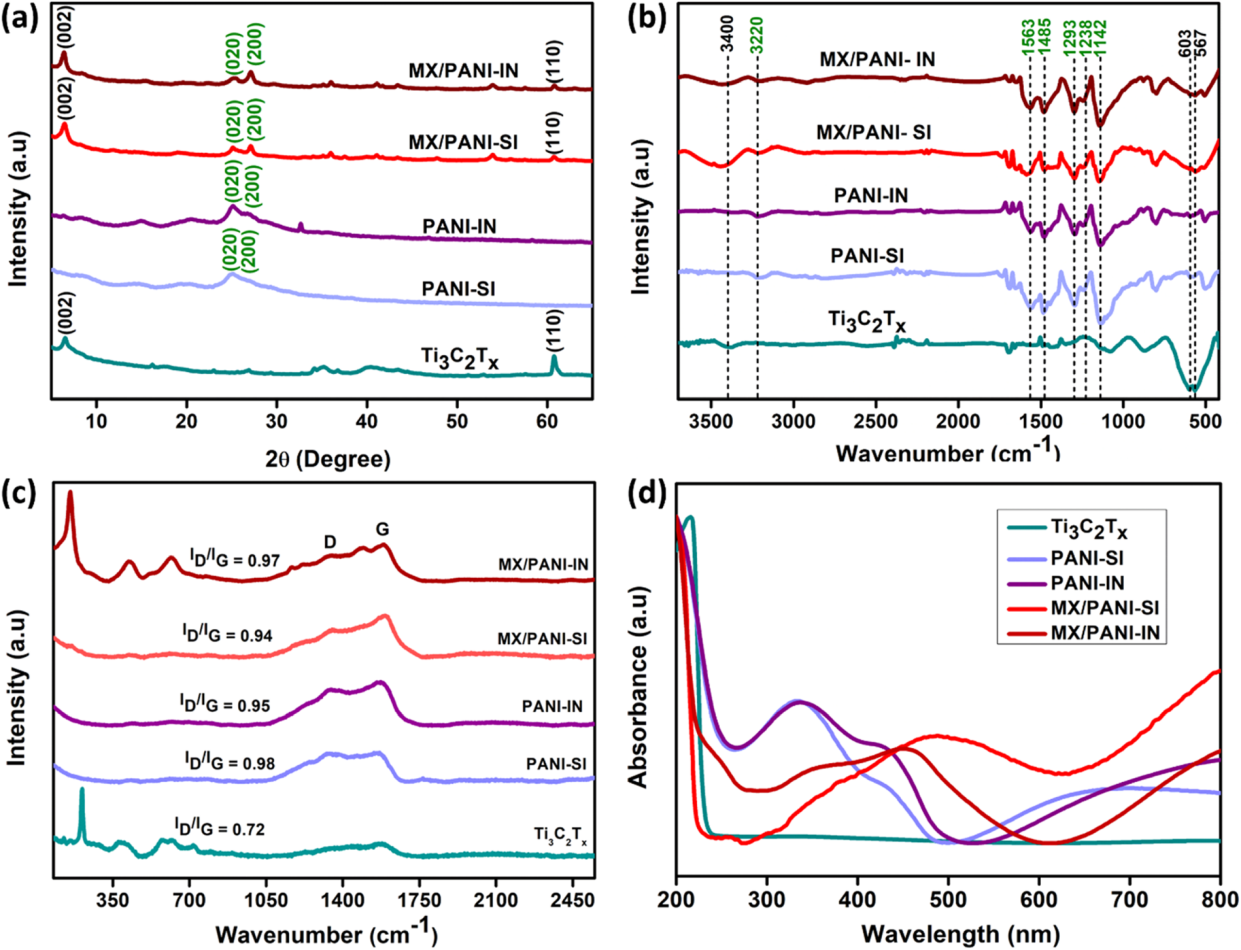
(a) XRD patterns; (b) FTIR spectra; (c) Raman spectra; and (d) UV-visible spectra of Ti_3_C_2_T_*x*_ MXene, PANI-SI, PANI-IN, MX/PANI-SI and MX/PANI-IN.

The standard XRD patterns for the Ti_3_AlC_2_ MAX phase and Ti_3_C_2_T_*x*_ MXene samples are illustrated in Fig. S6.† Notably, the primary diffraction peak (002) of Ti_3_AlC_2_ appears at 9.49°, whereas the peak for Ti_3_C_2_T_*x*_ is observed at a lower angle of 6.58°, indicating an increase in *c* parameters.^67^ The XRD patterns confirm the effective removal of Al layers and the substitution of Al with –F and –OH/=O terminating groups. Furthermore, the complete diminishing of the peak at 38.9° suggests the successful etching of the Al layers from the original Ti_3_AlC_2_ material.^2,67^ In Fig. 3a, the XRD pattern of Ti_3_C_2_T_*x*_ MXene shows two characteristic diffraction peaks at 6.58° and 60.76°, which correspond to the (002) and (110) planes of MXene, respectively, consistent with the XRD results of Ti_3_C_2_T_*x*_.^67,68^ Similarly, the diffraction pattern of pristine PANI shows distinct peaks of (020) and (200) at 20.38° and 25.17°, respectively.^69^ Furthermore, the successful formation of MXene/PANI composites by the interface and single-phase polymerisation methods is substantiated by the presence of reflections of both Ti_3_C_2_T_*x*_ MXene and PANI in MX/PANI-IN and MX/PANI-SI, as shown in Fig. 3a. The characteristic peak of Ti_3_C_2_T_*x*_ (002) downshifts from 6.54° to 6.47° in MX/PANI-SI and 6.54° to 6.41° in MX/PANI-IN, indicating the increased *d*-spacing after PANI intercalation (Fig.S7†). The MX/PANI-IN sample demonstrates better exfoliation following PANI intercalation in L/L interface-assisted polymerisation, as evidenced by the downshift of 2*θ*.

Fourier transform infrared (FTIR) spectroscopy was performed to assess the successful formation of the MXene/PANI composites. Fig. 3b presents the FTIR spectra of Ti_3_C_2_T_*x*_ MXene and MXene/PANI nanocomposites. The characteristic peaks of Ti_3_C_2_T_*x*_ MXene are located at 1659 cm^−1^, 1079 cm^−1^, 603 cm^−1^, and 567 cm^−1^, corresponding to C=O, C–O, Ti–O, and O–Ti–O, respectively.^70,71^ The broad peak at 3400 cm^−1^ corresponds to the O–H vibrational stretch of Ti_3_C_2_T_*x*_ MXene.^72^ Moving to the FTIR spectra of MXene/PANI composites (MX/PANI-IN and MX/PANI-SI), the peaks at 1485 cm^−1^ and 1563 cm^−1^ are ascribed to the C=C stretching vibrations of the quinoid and benzenoid ring of PANI.^73,74^ The peak at 1293 cm^−1^ and 1238 cm^−1^ corresponds to the C–N stretching frequencies of the benzenoid ring in polyaniline.^75^ The peak at 1142 cm^−1^ is due to the in-plane bending vibration of the C–H bond in the quinoid structure, and the broad band centered at 3220 cm^−1^ is due to the N–H stretching frequency. Apart from characteristic PANI peaks, the additional peaks in the 567–603 cm^−1^ range correspond to O–Ti–O and Ti–O vibrations, confirming the co-existence of Ti_3_C_2_ and PANI. Besides, the blueshifts of –OH peaks in FTIR spectra of MXene/PANI composites suggest the formation of stronger hydrogen bonding between MXene and the polymer network, thereby confirming the successful formation of MXene/PANI composites.

The as-prepared samples were further analyzed by confocal Raman spectroscopy, as displayed in Fig. 3c. The Raman spectra of MX/PANI-IN samples revealed two peaks at 1351 cm^−1^ and 1594 cm^−1^, which are indicative of the D-band associated with disordered carbon and the G-band linked to sp^2^-hybridized graphitic carbon atoms, respectively.^69^ Comparably, the MX/PANI-SI samples exhibited notable peaks at 1354 cm^−1^ and 1591 cm^−1^. The Raman spectra of Ti_3_C_2_T_*x*_ displayed distinct peaks in the range 210 to 407 cm^−1^ and are ascribed to the in-plane vibrations of surface groups attached to Ti atoms. The region from 566 to 744 cm^−1^ is assigned to carbon vibrational modes (both E_g_ and A_1g_).^50^ Raman spectra of MXene/PANI-IN exhibited hybrid peaks of PANI and Ti_3_C_2_T_*x*_, which proved that the Ti_3_C_2_T_*x*_ nanosheets were successfully decorated by PANI nanostructures. The *I*_D_/*I*_G_ ratio in the composite increases due to the hydrogen bonding and electrostatic interactions between Ti_3_C_2_T_*x*_ nanosheets and PANI. Compared to that of Ti_3_C_2_T_*x*_ (0.72) and PANI-IN (0.95), the intensity ratio (*I*_D_/*I*_G_) of MX/PANI-IN (0.97) was increased, suggesting that the PANI nanostructures on the MXene surface increased more defect sites owing to the bonding of heteroatoms (N atoms of PANI) with sp^2^ hybridized C atoms of Ti_3_C_2_T_*x*_.^76^ Meanwhile, MX/PANI-SI (0.94) has a lower *I*_D_/*I*_G_ ratio compared to PANI-SI (0.98), which might be due to the occurrence of different polymerisation kinetics in the presence of MXene, resulting in fewer defects and weaker bonding interactions with MXene. Moreover, the aggregated globular-natured PANI-SI (0.98) exhibited a higher *I*_D_/*I*_G_ ratio than that of tubular-natured PANI-IN (0.95). The Raman data also suggest that the L/L interface-assisted synthesis might lead to more disordered carbon structures in MXene due to the higher extent of polymer MXene interactions, as compared to the bulk polymerisation method. PANI-SI, PANI-IN, MX/PANI-SI, and MX/PANI-IN samples after 12 hours of the polymerisation reaction were vacuum filtered, dried at room temperature and redispersed in water to collect the UV-visible spectra (Fig. 3d). The characteristic peaks of PANI are observed at 333 nm and 429 nm for pure PANI samples, which arise from the π–π* and n–π* electronic transition within the benzenoid segments.^77^ The broad absorption peak in the range 600–800 nm observed for PANI-IN and PANI-SI arises from the π-polaron transition, suggesting the formation of the conducting emeraldine salt form of PANI in the presence of oxidant.^46,78,79^ Apart from this, additional peaks are observed for MX/PANI-IN at 359 nm, 458 nm, and 800 nm, and for MX/PANI-SI, a broad peak is observed at 487 nm and towards 800 nm, suggesting the completion of the polymerisation reaction. For MXene, the peak at 215 nm corresponds to the oxidation state of titanium in MXene, and a red shift is observed in this peak to 254 nm for both composites, suggesting the integration of polymer with MXene.

**Fig. 4:**
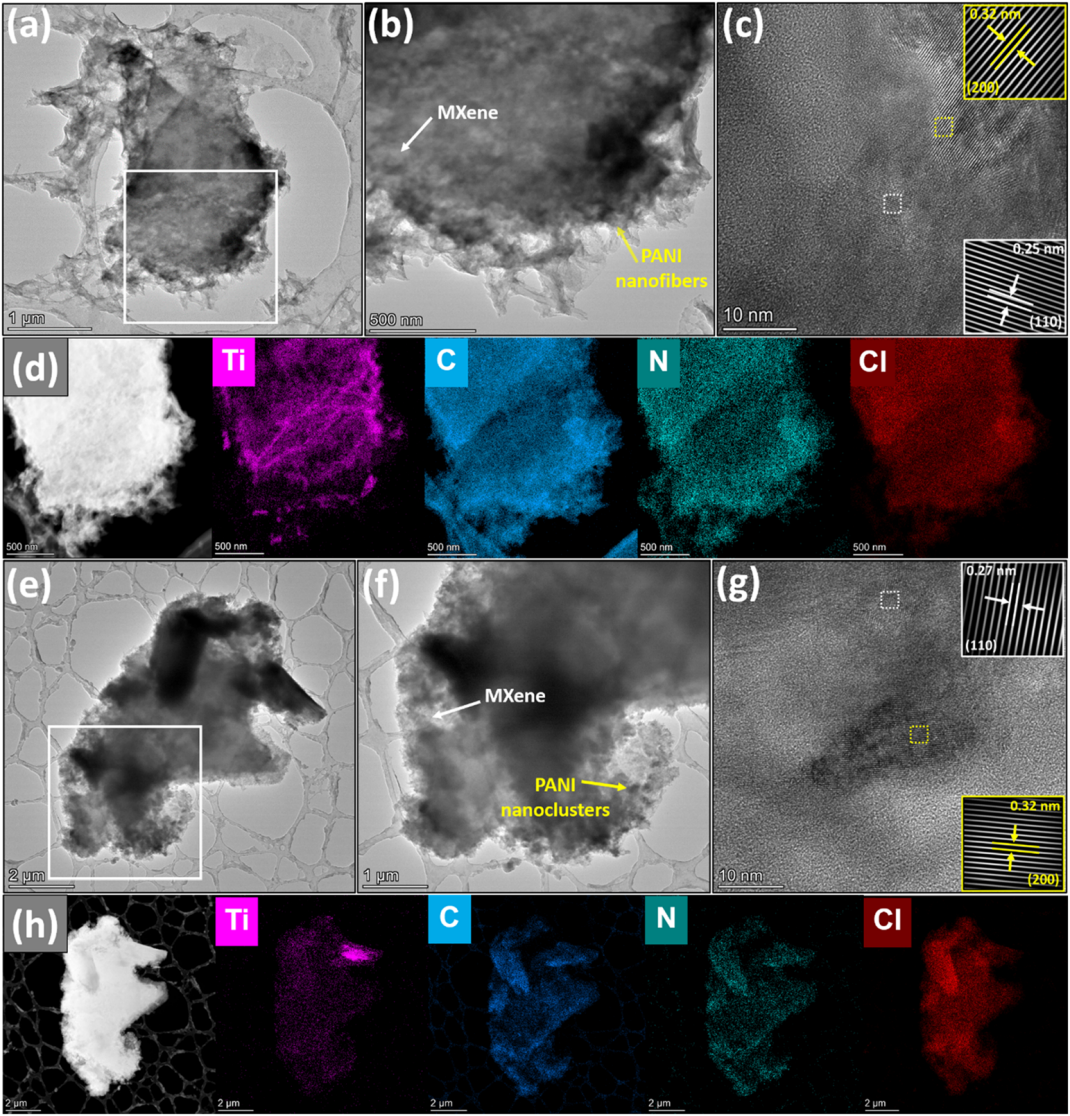
(a) The TEM image of MX/PANI-IN; (b) magnified HR-TEM image of the area marked by a square box in (a), clearly depicting the nanofiber growth of PANI over the MXene surface; (c) *d*-spacings and the insets showing respective inverse FFT images of MXene (white color markings) and PANI (yellow color markings); (d) corresponding EDS elemental mapping images of MX/PANI-IN; (e) the TEM image of MX/PANI-SI; (f) magnified HR-TEM image of the area marked by a square box in (e) depicting the aggregated growth of PANI over MXene; (g) *d*-spacings and the insets showing respective inverse FFT images of MXene (white color markings) and PANI (yellow color markings); (h) corresponding EDS elemental mapping images of MX/PANI-SI.

The distinct morphology of PANI polymerised using L/L interface and single-phase strategies and its distribution over the MXene surfaces were analyzed using HR-TEM imaging techniques, as shown in Fig. 4. The HR-TEM image (Fig. 4a and b) of MX/PANI-IN clearly explains the tubular-like growth of PANI over MXene, unlike the TEM image of MX/PANI-SI (Fig. 4e and f) that depicts a cluster-like growth of PANI over MXene. The laminar structure of the as-synthesized composites may be indiscernible in the TEM images due to the overlay of PANI on the MXene surface. The orientation of MXene is laminar in MX/PANI-IN with layers of PANI fibres, and this arrangement helps to improve the electron transfer. The magnified high-resolution TEM images of both samples (Fig. 4c and g) of the MXene/PANI composite show an interplanar spacing of 0.32 nm corresponding to the (200) plane of PANI. To gain further structural insights into the distribution of PANI into the MXene structure, HR-TEM EDS mapping was used for both samples (Fig. 4d and h), and an even distribution of C and N of PANI and Ti of MXene was confirmed. Fig. S8a and b† represent the SAED patterns of each sample, and the rings with distinct diffraction spots relate to the MXene/PANI nanocomposites being polycrystalline.

**Fig. 5:**
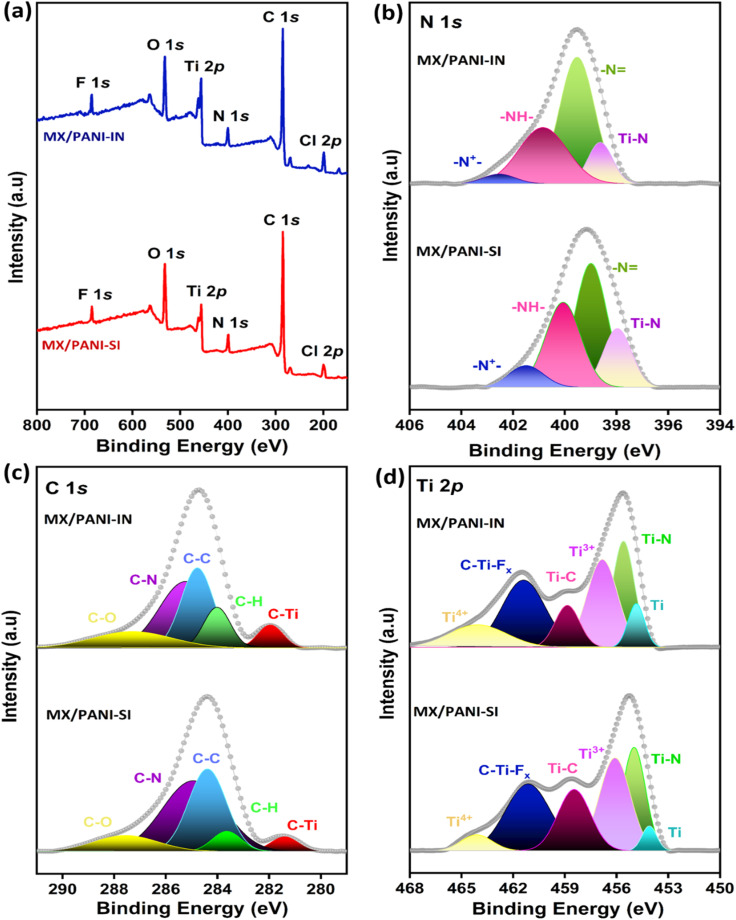
(a) XPS spectra: (a) survey scan and deconvoluted spectra of (b) nitrogen, (c) carbon and (d) titanium of MX/PANI-SI and MX/PANI-IN.

XPS spectra were measured to investigate the chemical constituents of Ti_3_C_2_T_*x*_ MXene and MXene/PANI nanocomposites synthesised. The survey scan and high-resolution XPS spectra of N 1s, C 1s and Ti 2p core levels of MX/PANI-SI and MX/PANI-IN are displayed in Fig. 5a–d, respectively. The survey spectrum and XPS analysis of Ti_3_C_2_T_*x*_ are provided in Fig. S9.† The survey scan of Ti_3_C_2_T_*x*_, MX/PANI-SI and MX/PANI-IN includes the common peaks of Ti, O, C, F and Cl. The presence of electronegative surface groups imparts an inherent negative charge to MXene, facilitating the adsorption of aniline radical cations necessary for the subsequent growth of polymer chains on its surface and interlayers.^45,80^ Compared with pristine Ti_3_C_2_T_*x*_, MX/PANI-SI and MX/PANI-IN contain additional N 1 s and more intense C 1s peaks in the survey scan, indicating the content of PANI.^64,81^ Furthermore, the greater intensity of the Cl peak in both samples compared to Ti_3_C_2_T_*x*_ indicates the presence of conductive doped PANI. The high-resolution XPS peak of N 1 s in MX/PANI-SI is fitted to four peaks which are assigned to –N^+^– (401.47 eV), –NH– (400.05 eV), –N= (398.97 eV) and Ti–N (397.92 eV). The high-resolution XPS peaks of N 1s in MX/PANI-IN are assigned to –N^+^– (401.50 eV), –NH– (400.05 eV), –N= (398.97 eV) and Ti–N (397.94 eV). These nitrogen functional groups demonstrate the interactions between PANI and Ti_3_C_2_T_*x*_ and are characteristic of the doped PANI.^66,82,83^ The C 1s peaks in MX/PANI-SI are deconvoluted into C–O (287.60 eV), C–N (285.10 eV), C–C (284.40 eV), C–H (283.60 eV), and C–Ti (281.40 eV), while those in MX/PANI-IN are deconvoluted into C–O (287.60 eV), C–N (285.20 eV), C–C (284.70 eV), C–H (283.90 eV) and C–Ti (281.90 eV).^84,85^ The Ti 2p peaks of MX/PANI-SI are fitted into six peaks, which are assigned to Ti^4+^ (464.10 eV), C–Ti-Fx (461.15 eV), Ti–C (458.46), Ti^3+^(456.06 eV), Ti–N (455 eV) and Ti (454.06 eV). The Ti 2p peaks of MX/PANI-IN are fitted to Ti^4+^ (464.06 eV), C–Ti-Fx (461.40 eV), Ti–C (458.82 eV), Ti^3+^(456.83 eV), Ti–N (455.5 eV) and Ti (454.80 eV).^86^ The peak fitting results are summarised in Table S2.†

### 3.2 Electrochemical characterization

**Fig. 6:**
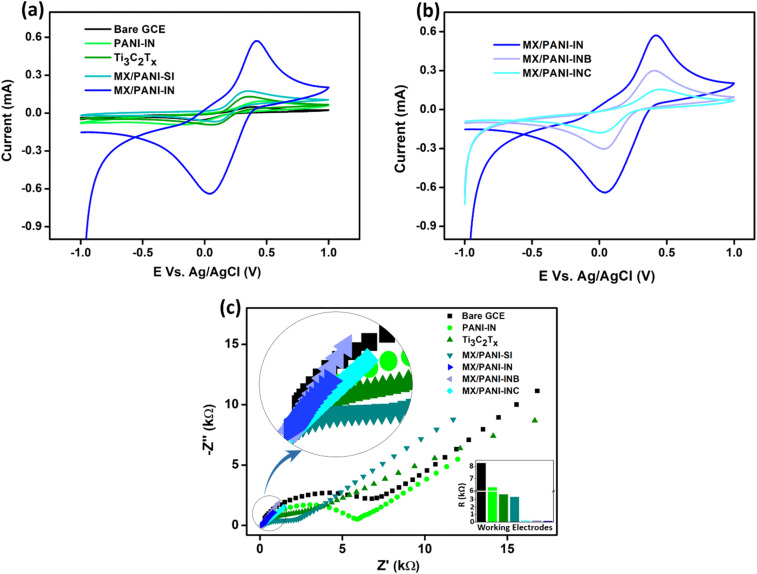
(a) The CV responses of various modified electrodes in 5 mM K_4_[Fe(CN)_6_]/0.1 M KCl; (b) CV responses of modified electrodes with varying ratios of PANI and MXene; (c) Nyquist plots of the bare GCE and different modified GCEs in the presence of 5 mM K_4_[Fe(CN)_6_] in 0.1 M KCl, with the inset showing the interfacial charge transfer resistance of the electrodes (*R*_ct_ values) obtained from the corresponding equivalent circuit. A magnified version at higher frequencies is also shown in the inset.

The electrochemical analysis of the glassy carbon electrode modified with Ti_3_C_2_T_*x*_ MXene, PANI-IN, and MXene/PANI composites was conducted by employing cyclic voltammetry (CV) analysis in a solution comprising 5 mM K_4_[Fe(CN)_6_] in 0.1 M KCl solutions. Fig. 6a illustrates the presence of a redox peak in all the samples, which originates from the reversible redox behaviour of [Fe(CN)_6_]^3−/4−^ species on the modified GCE. The highest current peaks observed for MX/PANI-IN confirm the enhanced electrocatalytic activity compared to bare GCE, PANI-IN, Ti_3_C_2_T_*x*_, and MX/PANI-SI. The amount of aniline introduced to the upper toluene layer in the interface-assisted polymerisation method was then systematically altered to 7 mmol and 9 mmol while retaining a constant amount of MXene, and these composites were labelled as MX/PANI-INB and MX/PANI-INC, respectively. The CV response of MX/PANI-INB and MX/PANI-INC was recorded and compared with that of MXene/PANI-IN. Fig. 6b shows that the composite labelled MXene/PANI-IN shows the highest electrochemical performance. The limited exposure of MXene active sites at higher loading on PANI in the composites could result in declined electrochemical performance. The Nyquist plots in Fig. 6c illustrate the reduced charge transfer resistance for the MX/PANI-IN modified GCE relative to other modified GCEs, resulting in enhanced electrochemical activity due to improved charge transfer. This is probably due to the enhanced electron conductivity, increased surface area, and the synergistic interaction between the Ti_3_C_2_T_*x*_ MXene nanosheets and PANI nanofibers. The equivalent circuit diagrams corresponding to the Nyquist plots are shown in Fig. S10.† To put it together, the *in situ* generation of MXene/PANI at the bisolvent interface outperforms, hence the MXene/PANI composite, referred to as MX/PANI-IN, was identified for detailed investigations for the electrochemical detection of biomarkers.

**Fig. 7:**
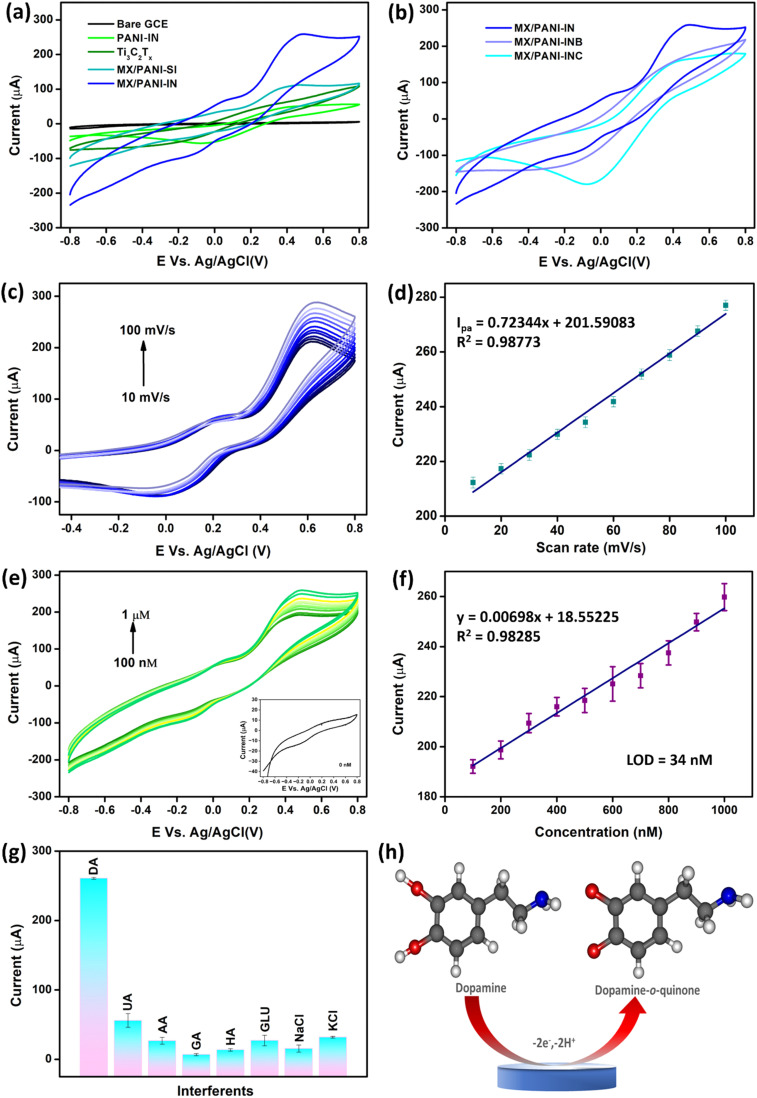
(a) Cyclic voltammograms for the comparison of current responses of the bare GCE with various modified electrodes towards 1 μM DA at 100 mV s^−1^; (b) cyclic voltammograms for the comparison of current responses of modified electrodes with varying ratios of polymer and MXene towards 1 μM DA at 100 mV s^−1^; (c) cyclic voltammograms of the MX/PANI-IN modified electrode towards 1 μM DA at varying scan rates; (d) calibration plot of anodic peak current *vs.* scan rate; (e) cyclic voltammograms of MX/PANI-IN modified electrodes in the presence of varying concentrations of DA in the range of 100 nM to 1 μM at pH 7 and scan rate 100 mV s^−1^; the inset shows the CV scan of the MX/PANI-IN modified electrode in the presence of 0 nM DA at pH 7 and scan rate 100 mV s^−1^, (f) calibration plot of anodic peak current *vs.* concentration; (g) bar plot showing selectivity of the MX/PANI-IN modified GCE towards DA in the presence of different interfering molecules such as UA, AA, GA, HA, GLU, NaCl and KCl; (h) possible mechanism of DA oxidation on the MX/PANI-IN modified GCE.

To analyse the operational electrochemical stability of Ti_3_C_2_T_*x*_ MXene and the MX/PANI-IN composite, CV analysis was performed over a series of 10 cycles in 0.1 M phosphate buffer (PB) solution, and the data are illustrated in Fig. S11.† The CV results show that there is no significant change in the current response for MX/PANI-IN, which confirms the improved operational stability of the MX/PANI-IN composite. This stability is attributed to the better synergic interaction between PANI nanofibers and MXene, induced as a result of controlled interfacial polymerisation growth. Ultimately, the electrochemical detection of DA using the GCE modified with MXene and MXene/PANI-IN composite was evaluated in a 0.1 M phosphate buffer (PB) solution (pH 7.0) containing 1 μM DA(Fig. 7a). Additionally, the same response evaluation was carried out for the composites with various ratios of MXene and PANI (Fig. 7b). Strikingly, MX/PANI-IN rendered a significantly raised anodic response (*I*_pa_ ∼258.48 μA) with a well-resolved oxidation peak at a potential of 0.48 V and was therefore selected for further electrochemical investigations. The reverse scan, however, does not show a matching cathodic current response for reduction, indicating that the DA has irreversibly undergone electrochemical oxidation. The enhanced activity of the MX/PANI-IN composite might be due to the optimum concentration of tubular PANI in the Ti_3_C_2_T_*x*_ matrix to get an enhanced electrochemical transfer response. To assess the scan rate-dependent electrocatalytic behaviour of DA oxidation on the MX/PANI-IN-modified GCE, cyclic voltammograms were obtained in 0.1 M PB solution containing 1 μM DA at varying scan rates. The CV analysis data given in Fig. 7c depict an increase in peak current as the scan rate escalates from 10 to 100 mV s^−1^. The relationship between the peak current density and the scan rate is illustrated in Fig. 7d, revealing a linear curve with an *R*^2^ value of 0.98773. This behaviour indicates that the electrocatalytic oxidation of DA on the MX/PANI-IN-modified GCE follows a typical adsorption-controlled electron transfer process. The CV curves depicting the oxidation of DA at various concentrations on the MX/PANI-IN-modified GCE are presented in Fig. 7e. The background CV curve of the MX/PANI-IN-modified electrode in 0 nM DA is depicted as an inset in Fig. 7e. The oxidation peak current demonstrates a linear increase over a concentration range of 100 nM–1 μM. The calibration curve was plotted between peak current and concentration of DA, which is depicted in Fig. 7f. The calibration curve exhibits a linear relationship with a promising regression constant of 0.98285. Furthermore, the limit of detection (LOD) was calculated using the 3 sigma method, and the value of LOD was found to be 34 nM. To assess the selectivity of the proposed sensor with other electroactive analytes, we observed the activity of the MX/PANI-IN modified GCE sensor for DA detection in the presence of other analytes using CV. We compared the activity toward the detection of DA by taking the interfering analytes uric acid (UA), ascorbic acid (AA), glutamic acid (GA), histamine (HA), glucose (GLU), NaCl and KCl at a concentration 10 times higher than that of DA. As shown in Fig. 7g, the selectivity analysis shows the highest current response for DA on the MX/PANI-IN-modified GCE compared to the interfering molecules, which confirms the promising selectivity of the proposed sensor for the detection of DA. The CV curves corresponding to the selectivity analysis are given in Fig. S12.† The summary of various MXene-based electrochemical sensors for DA detection reported recently is given in Table S3.†Fig. 7h depicts the electrochemical oxidation mechanism of DA at the MX/PANI-IN-modified GCE. In this process, DA undergoes two-electron, two-proton oxidation to form dopamine-*o*-quinone.

## 4. Conclusions

In the present work, we have explored the physicochemical tuning of the MXene/PANI nanocomposite using two distinct synthesis strategies: single-phase polymerisation and interfacial *in situ* polymerisation. When compared to the MXene/PANI composite generated *via* single-phase polymerisation, the interfacial approach produced a distinctive fibre-like PANI decorated morphology and improved exfoliation of MXene layers while keeping the other reaction parameters unchanged. To the best of our knowledge, interface-assisted synthesis of *in situ*-generated MXene/PANI composite *via* oxidative chemical polymerisation is not reported. Intriguingly, the solubility constraints of the oxidant and monomer in a single solvent phase were eliminated by the wise choice of an aqueous/organic interface in tuning the morphology and properties at a nano level. UV-visible spectroscopy was employed to monitor the development of reactant consumption and product production in the corresponding organic/aqueous phases, demonstrating controlled polymerisation at the L/L interface. The regulated release of reactants *via* the interface avoids unwanted side-chain branching reactions, resulting in the *in situ* formation of long-chain polymers. Considering all aspects of structural and electrochemical characterization studies, it is evident that the MXene/PANI interface (MX/PANI-IN) is a superior electrode material for electrochemical sensors to detect DA levels up to a limit of detection of 34 nM. Furthermore, the reported interfacial *in situ* polymerisation strategy could be adapted to the successful exfoliation of various 2D nanostructures in a simple and efficient manner.

## Conflicts of interest

The authors declare no conflict of interest.

## Supplementary Material

NA-007-D5NA00374A-s001

## Data Availability

The data supporting this article have been included as part of the ESI.†
