# Supplementary material for: Liquid/liquid interface assisted in situ polymerisation of aniline on Ti3C2Tx MXene for electrochemical detection of dopamine
Source: Nanoscale Adv. 2025 Jul 8;7(16):4980–93. doi: 10.1039/d5na00374a (PMC12235557; doi:10.1039/d5na00374a)
Supplement: NA-007-D5NA00374A-s001 [file NA-007-D5NA00374A-s001.pdf]

Supplementary Information (SI) for Nanoscale Advances.
This journal is © The Royal Society of Chemistry 2025

Supporting Information

Liquid/Liquid interface assisted *in situ* polymerisation of aniline on $Ti_3C_2T_x$ MXene for electrochemical detection of dopamine

Anjali S[a], Aiswarya A.S[a], Athul Beena Radhakrishnan[a], Mini Mol Menamparambath [a]*

[a] Department of Chemistry, National Institute of Technology Calicut, Calicut-673601, Kerala, India.

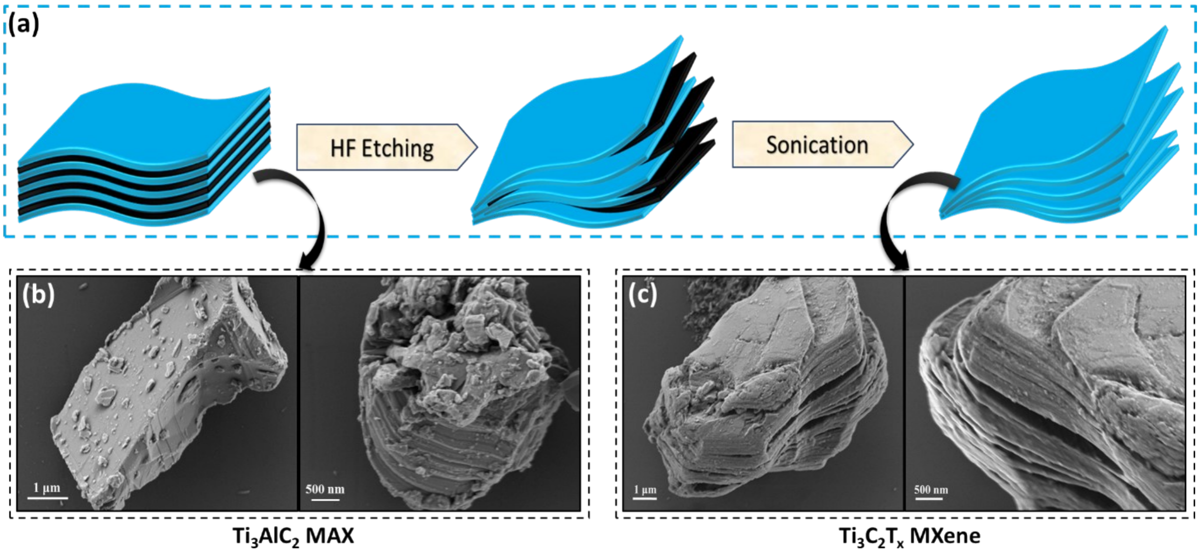

Supporting Fig. S1. (a) Schematics of synthesis route for MXene from MAX phase by HF etching method ; (b) SEM images of Ti_3AlC_2 MAX phase; (c) SEM images of $Ti_3C_2T_x$ MXene

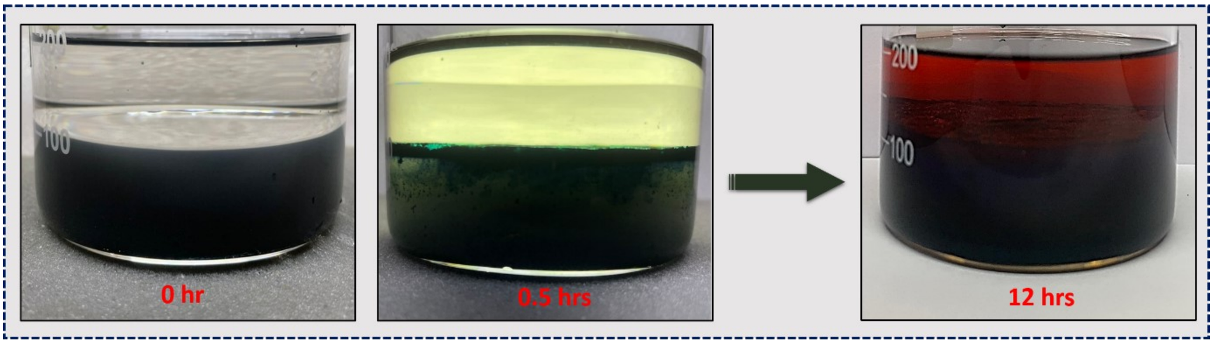

(a) L/L interface-assisted polymerisation synthesis of MXene/PANI

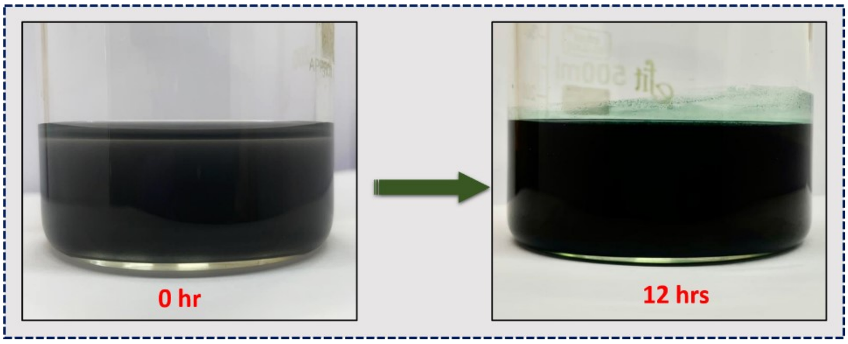

(b) Single-phase polymerisation synthesis of MXene/PANI

Supporting Fig. S2. (a) MXene/PANI synthesis by interfacial polymerisation. Formation of MXene/PANI at the L/L interface (toluene/water) at initial stages and the products moved to the lower aqueous layer at the end of the reaction. (b) MXene/PANI synthesis by single-phase (bulk) polymerisation in the aqueous phase.

Sl. No.	Sample code	Density of MXene Dispersion added	Volume of Toluene layer	Volume of Aqueous layer	Amount of Aniline added	Ratio of Aniline: APS
1	MX/PANI-SI	0.1 g/mL	NIL	100 mL	5 mmol	1:1
2	MX/PANI-IN		100 mL		5 mmol	
3	MX/PANI-INB		100 mL		7 mmol	
4	MX/PANI-INC		100 mL		9 mmol	
5	PANI-SI	No MXene added	NIL		5 mmol	
6	PANI-IN	No MXene added	100 mL		5 mmol	

Supporting Table S1. Table listing the sample code, and the corresponding synthesis parameters of the MXene/PANI composites and pure PANI synthesised using the interface and single-phase polymerisation. *'IN' indicates Interface and 'SI' indicates Single-phase*

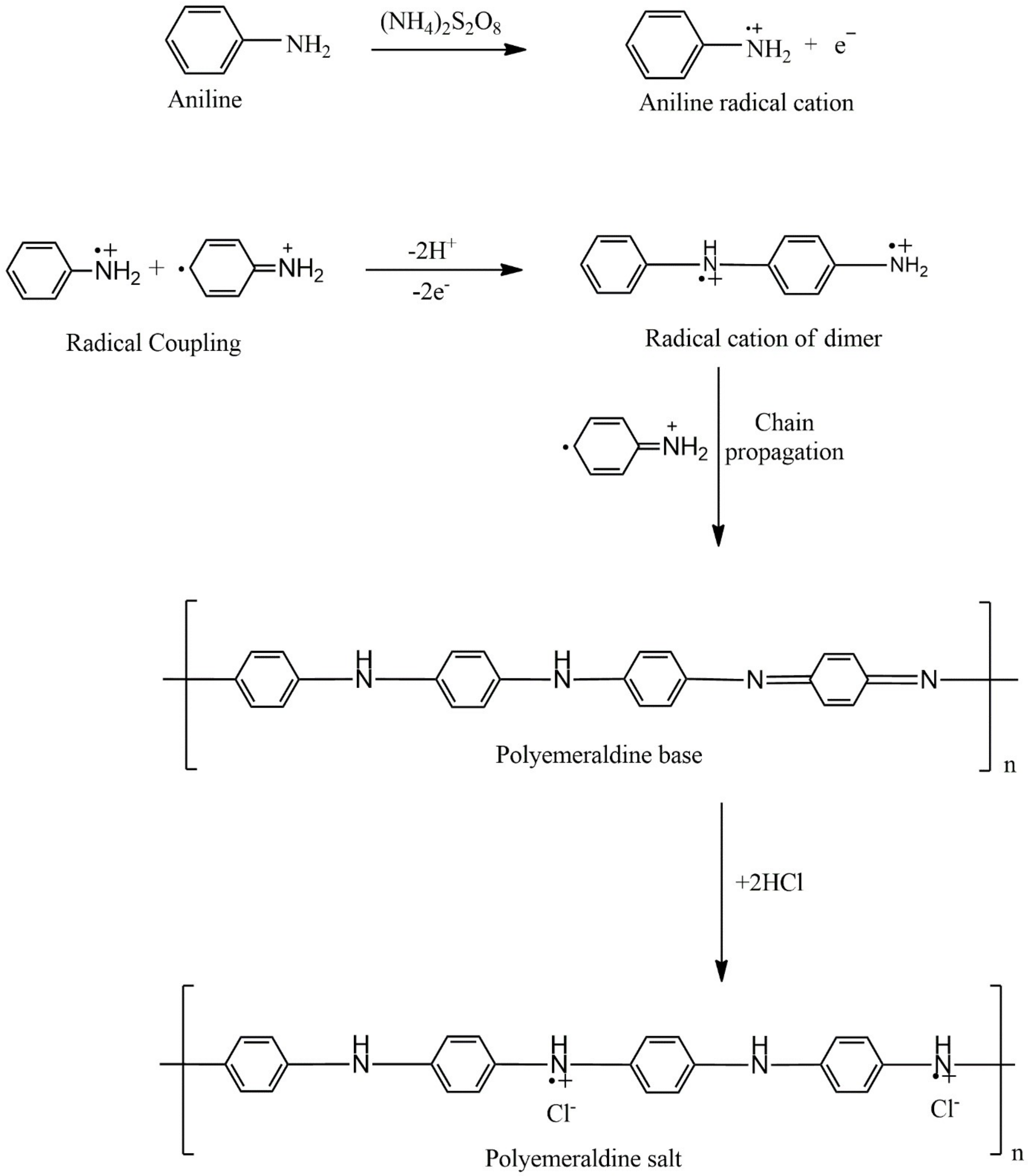

Supporting Fig. S3. Mechanism of polymerisation of aniline to polyaniline.

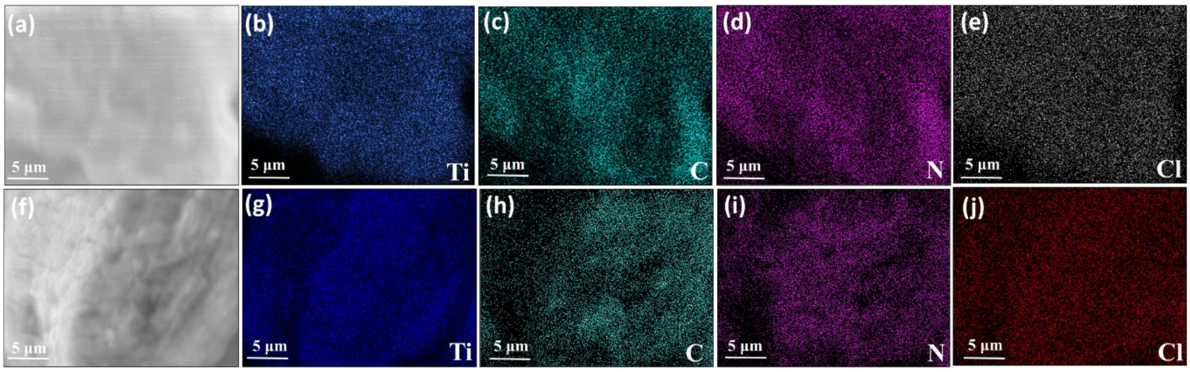

Supporting Fig. S4. SEM-EDS mapping images of the MXene/PANI nanocomposite synthesised by (a) to (e) interface-assisted polymerisation and (f) to (j) single-phase polymerisation method.

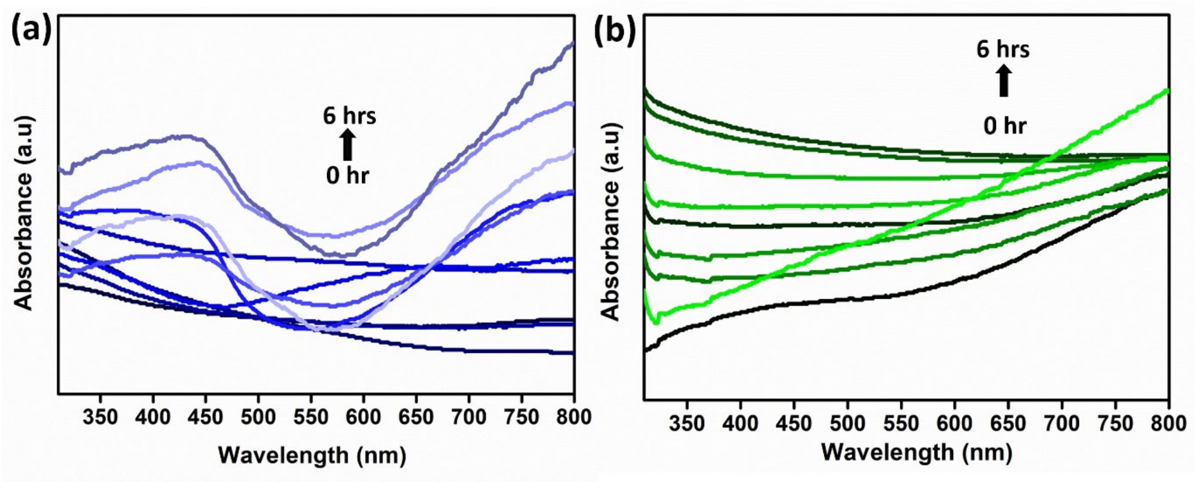

Supporting Fig. S5. (a & b) Enlarged UV–Visible spectra within the wavelength range of 350 to 800 nm presented in Fig. 2(b) & 2(c) of the main manuscript respectively.

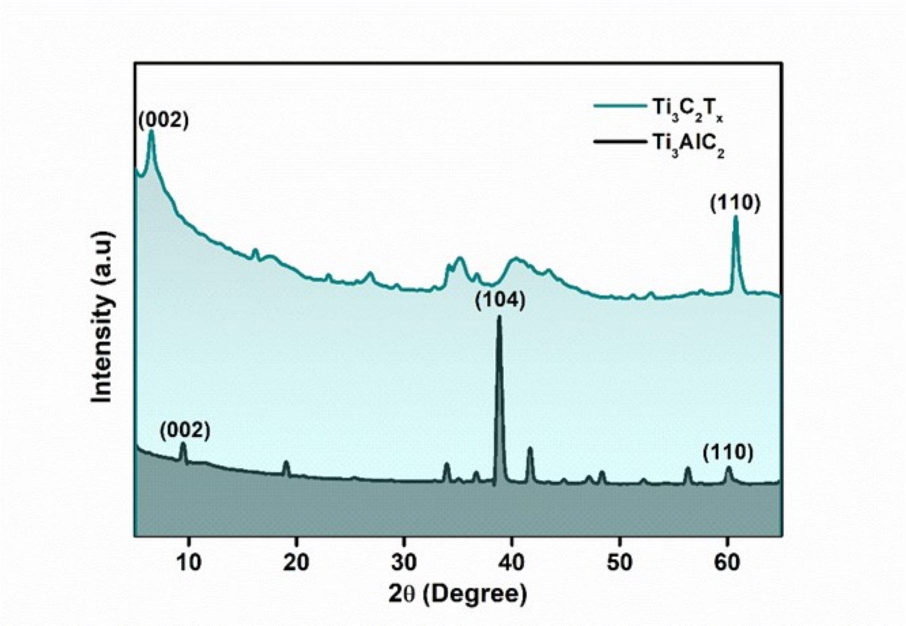

Supporting Fig. S6. XRD patterns of the synthesised Ti_3AlC_2 MAX and $Ti_3C_2T_x$ MXene.

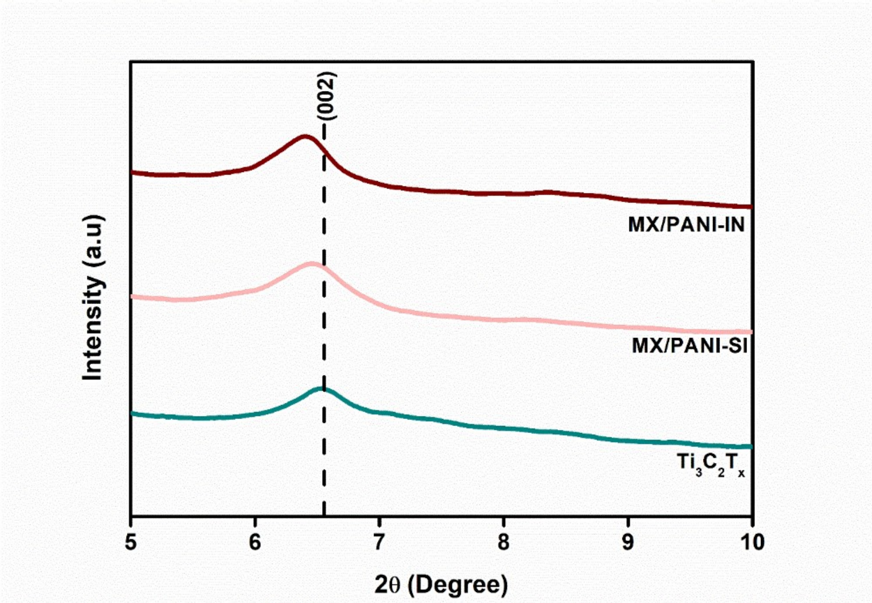

Supporting Fig. S7. Magnified XRD pattern in the range 5 to 10° depicting the shift in (002) peak in MXene/PANI composites.

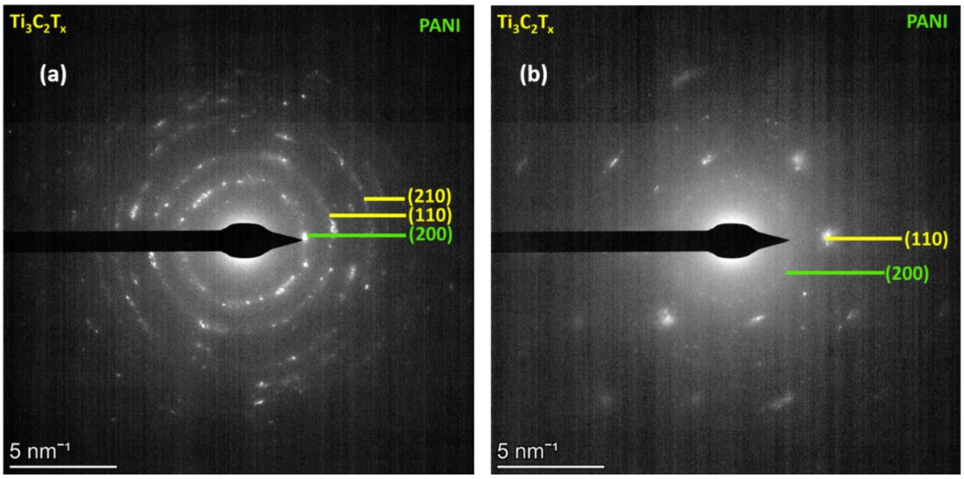

Supporting Fig. S8. SAED pattern corresponding to the lattice planes of (a) MX/PANI-IN and (b) MX/PANI-SI.

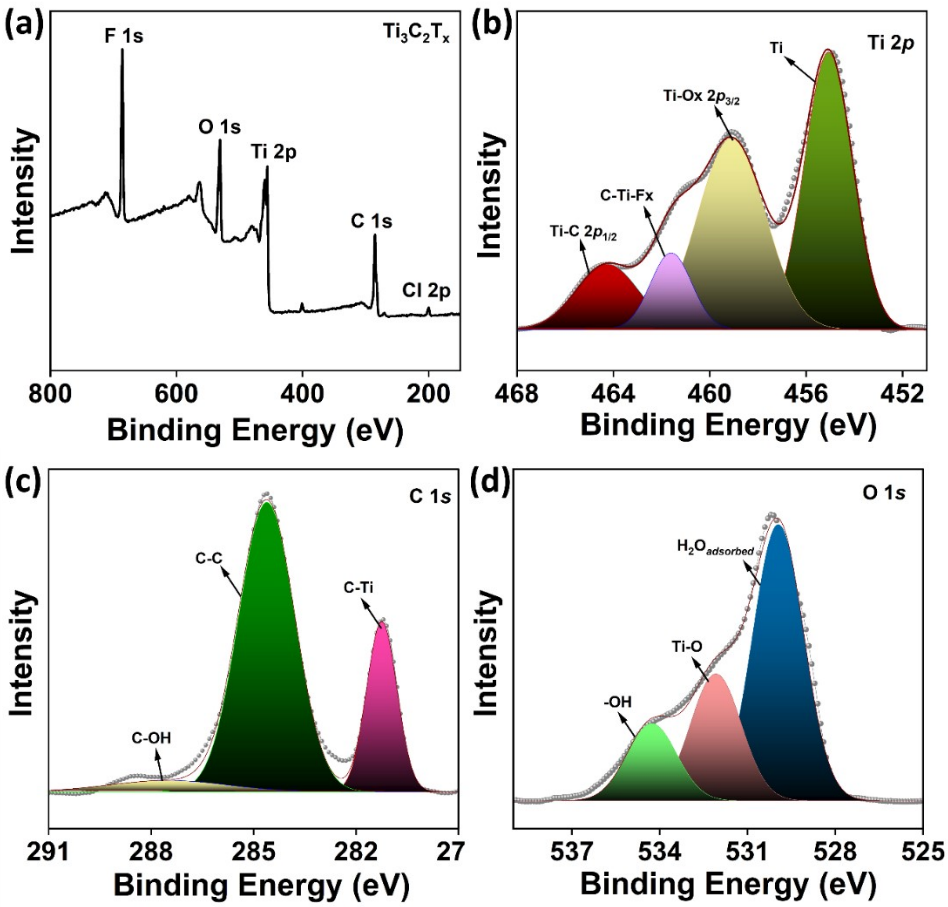

Supporting Fig. S9. XPS spectra (a) survey scan and deconvoluted spectra of (b) titanium, (c) carbon and (d) oxygen of $Ti_3C_2T_x$ MXene.

Sl. No.	Sample Code	Region	Binding Energy (eV)	Assigned to
1	$Ti_3C_2T_x$	Ti 2*p*	464.21<br>461.60<br>459.12<br>455.08	Ti-C<br>C-Ti-F_x<br>TiO_x<br>Ti
		C 1*s*	287.50<br>284.60<br>281.22	C-OH<br>C-C<br>C-Ti
		O 1*s*	534.24<br>532.07<br>529.92	-OH<br>Ti-O<br>H_2O adsorbed
2	MX/PANI-SI	N 1*s*	401.50<br>400.05<br>398.97<br>397.92	–N^+-<br>-NH-<br>-N=<br>Ti-N
		C 1*s*	287.60<br>285.10<br>284.40<br>283.60<br>281.40	C-O<br>C-N<br>C-C<br>C-H<br>C-Ti
		Ti 2*p*	464.10<br>461.15<br>458.46<br>456.06<br>455.00<br>454.06	Ti^{4+}<br>C-Ti-F_x<br>Ti-C<br>Ti^{3+}<br>Ti-N<br>Ti
3	MX/PANI-IN	N 1s	401.50<br>400.05<br>398.97<br>397.94	–N^+-<br>-NH-<br>-N=<br>Ti-N
		C 1*s*	281.90<br>283.90<br>284.70<br>285.20<br>287.60	C-O<br>C-N<br>C-C<br>C-H<br>C-Ti
		Ti 2*p*	464.06<br>461.42<br>458.82<br>456.83<br>455.50<br>454.80	Ti^{4+}<br>C-Ti-F_x<br>Ti-C<br>Ti^{3+}<br>Ti-N<br>Ti

Supporting Table S2. XPS peak fitting results for $Ti_3C_2T_x$, MX/PANI-SI and MX/PANI-IN.

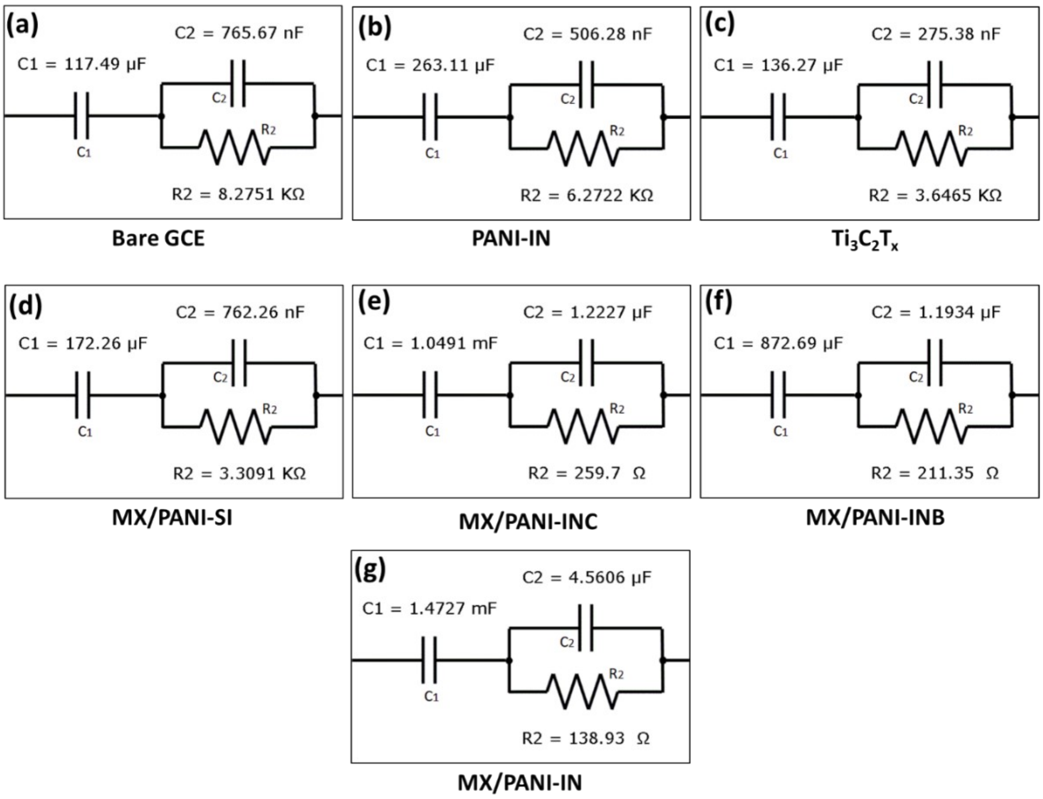

Supporting Fig. S10. The equivalent circuit diagrams corresponding to the Nyquist plots shown in Fig. 4a.

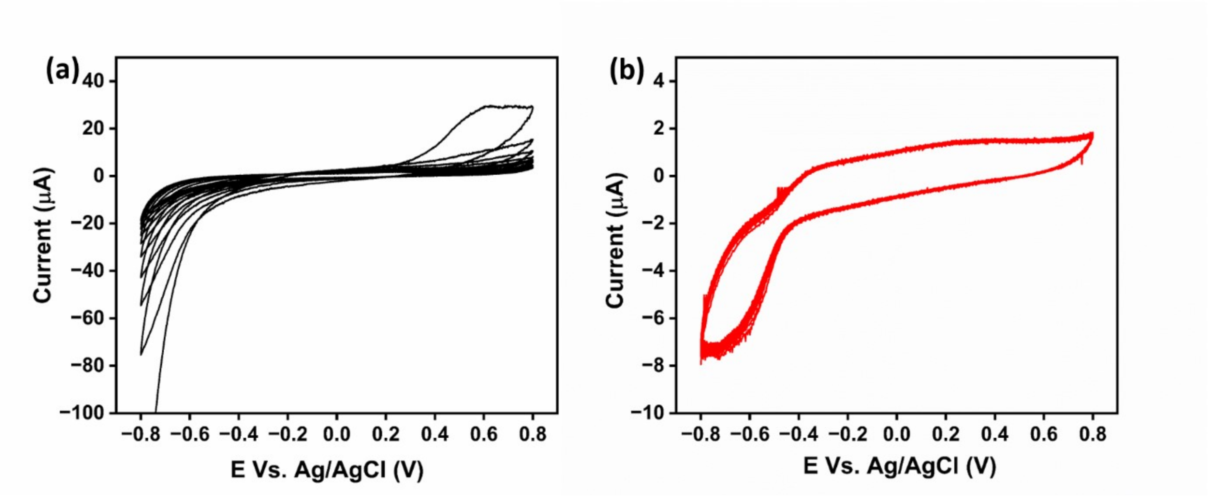

Supporting Fig. S11. Continuous CV scans consisting of 10 cycles of (a) $Ti_3C_2T_x$ MXene and (b) MX/PANI-IN for comparing the operational electrochemical stability

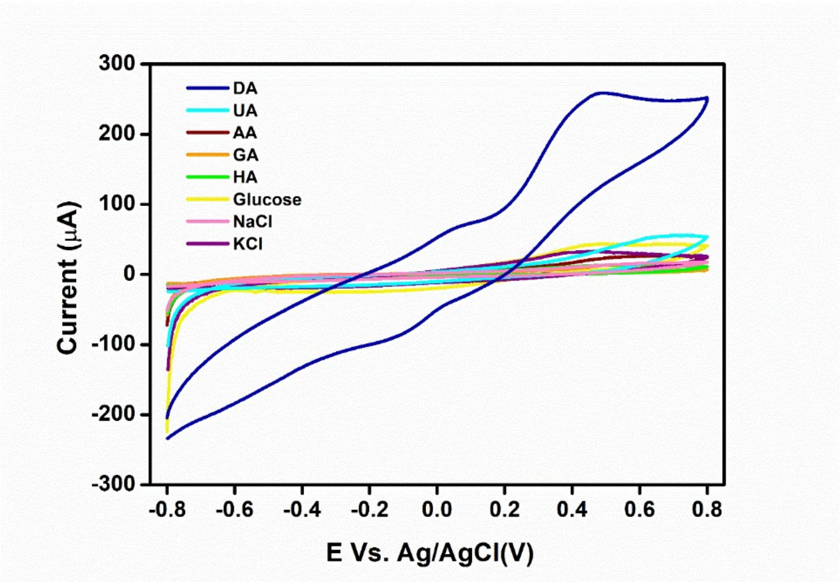

Supporting Fig. S12. CV scans for different interfering analytes at 10 times higher concentrations than dopamine.

Electrode Material	Analyte	Method	Linear Range	LOD	Ref.
SnO_2 QD@Ti_3C_2	DA	CV	0.004–8.0 μM	0.002 μM	[1]
Ti_3C_2/G-MWCNT/ZnO	DA	DPV	0.01–30 μM	3.2 nM	[2]
(V 0.05) K^+-$Ti_3C_2T_x$	DA	DPV	1–10 μM	8.4 nM	[3]
Ti_3C_2/DNA/Pd/Pt	DA	Amperometry	0.2–1000 μM	30 nM	[4]
Ti_3C_2/holey graphene	DA	DPV	0.5−50 μM	0.06 μM	[5]
ZnO/$Ti_3C_2T_x$/nafion/Au	DA	Amperometry	0.1−1200 μM	0.076 μM	[6]
MOF–Ti_3C_2	DA	DPV	0.09–0.13 μM	110 nM	[7]
PDI–MXene	DA	Amperometry	100–1000 μM	240 nM	[8]
Carbon fiber paper–MXene–MoS_2	AA, DA,	DPV	10-1000 μM (AA)	0.89 μM	[9]

	UA and miRNA		0.5-200 µM (DA)<br><br>0.5-150 µM (UA)<br>0.1 fM ~10 fM<br>10 fM ~ 10 nM (miRNA)	(AA), 0.23 µM (DA), and 0.35 µM (UA), miRNA (3.16 aM)	
$Ti_3C_2T_x$/PPy	DA and UA	DPV	12.5–125 µM (DA), 50–500 µM (UA)	0.37 µM (DA), 0.15 µM (UA)	[10]
**$Ti_3C_2T_x$/PANI**	**DA**	**CV**	**100 nM – 1 µM**	**34 nM**	**This work**

Supporting Table S3. The summary of various MXene-based electrochemical sensors for DA detection reported recently

References:

1 Y. Shi, K. Hu, L. Mei, X. Yang, Y. Shi, X. Wu, X. min Li, M. Miao and S. Zhang, *Microchim. Acta*, 2022, **189**, 1–10.

2 M. Ni, J. Chen, C. Wang, Y. Wang, L. Huang, W. Xiong, P. Zhao, Y. Xie and J. Fei, *Microchem. J.,* 2022, **178**, 107410.

3 J. Ramadoss, A. Sonachalam, K. Yusuf and M. Govindasamy, *Mikrochim. Acta*, 2024, **191**, 613.

4 J. Zheng, B. Wang, A. Ding, B. Weng and J. Chen, *J. Electroanal. Chem.*, 2018, **816**,

189–194.

5 Y. Zhang, L. Zhang, C. Li, J. Han, W. Huang, J. Zhou and Y. Yang, *Microchem. J.*,2022,**181**, 107713.

6 M. Cao, S. Liu, S. Liu, Z. Tong, X. Wang and X. Xu, *Microchem. J.*,2022, **175**,107068.

7 J. Paul and J. Kim, *Appl. Surf. Sci.,* 2023, **613**, 156103.

8 U. Amara, M. T. Mehran, B. Sarfaraz, K. Mahmood, A. Hayat, M. Nasir, S. Riaz and M. H. Nawaz, *Mikrochim. Acta.*,2021, **188**, 230.

9 J. Zhao, C. He, W. Wu, H. Yang, L. Peng, L. Wen and Z. Hu, *Chemical Engineering Journal,*2022, **446,** 136841.

10 Q. You, Z. Guo, R. Zhang, Z. Chang, M. Ge, Q. Mei and W. Dong, *Sensors*, 2021, **21**, 3069.
